# The Role of Controllability and Foreseeability in Children's Counterfactual Emotions

**DOI:** 10.1111/cdev.14224

**Published:** 2025-02-14

**Authors:** Alicia K. Jones, Shalini Gautam, Jonathan Redshaw

**Affiliations:** ^1^ School of Psychology The University of Queensland Brisbane Queensland Australia; ^2^ Department of Psychology Harvard University Cambridge Massachusetts USA

**Keywords:** counterfactual emotions, counterfactual thinking, regret

## Abstract

Counterfactual emotions such as regret may aid future decision‐making by encouraging people to focus on controllable features of personal past events. However, it remains unclear when children begin to preferentially focus on controllable features of such events. Across two studies, Australian 4–9‐year‐olds (*N* = 336, 168 females; data collected during 2021–2022) completed tasks that led to positive or negative personal outcomes, and then reported their emotions toward different aspects of these tasks. In both studies, younger children unexpectedly reported stronger sadness toward uncontrollable or unforeseeable aspects of negative events, and only by 8–9 years did many children report stronger sadness toward controllable or foreseeable aspects. The tendency to focus on more functional counterfactuals may therefore emerge relatively late in development.

The capacity to consider alternative versions of events that have already occurred is a complex form of *counterfactual thinking* (Beck and Riggs 2014; Epstude and Roese 2008; Kushnir 2022; Rafetseder and Perner 2012). After failing an exam, for instance, you may consider what else could have happened before or during the exam to improve your performance. Although it is often possible to consider many different counterfactual versions of a given past event, the functional theory of counterfactual thinking posits that reflecting on counterfactuals that were within our control (rather than counterfactuals that were out of our control) is more beneficial for learning to behave adaptively in the future (Roese and Epstude 2017). If you want to prevent yourself failing other exams in the future, for example, it would be more beneficial to consider an aspect of the past that you had control over (e.g., “If I had not procrastinated, if I had implemented effective study methods, or if I had scheduled enough time to study, then I would have passed my exam”) than an aspect of the past that was out of your control (e.g., “If the exam had been scheduled at a later time, if the exam questions had been easier, or if I had been assigned a different lecturer, then I would have passed my exam”).

Studies have widely investigated the role of controllability in adults' counterfactual thinking. Some studies have tasked adults with generating counterfactuals that another person might consider following a described event (Frosch et al. 2015; Girotto et al. 2007; Maloney and Egan 2017), whereas others have tasked participants with generating counterfactuals about personal past choices that have led to a negative outcome (Markman et al. 1995). Another common method is to first expose participants to a difficult task (e.g., *multiply 69 × 73 in 30 seconds*), and then, after they inevitably fail to complete the task, prompt them to consider counterfactuals (Ferrante et al. 2013; Girotto et al. 2007; Maloney and Egan 2017; Mercier et al. 2017; Stragà et al. 2022). Consistent with the functional theory of counterfactual thinking, many studies find that adults are more likely to report controllable counterfactuals than uncontrollable counterfactuals when asked to reflect on someone else's performance, on their own past choice, or task failure (Frosch et al. 2015; Girotto et al. 2007; Maloney and Egan 2017; Markman et al. 1995). Indeed, Maloney and Egan's (2017) further found that adults who reported controllable counterfactuals were significantly more likely to improve their performance in a subsequent task than those who generated uncontrollable counterfactuals. By contrast, there is some evidence that adults predominantly consider controllable aspects of imagined events only when specifically prompted to think of the future (Ferrante et al. 2013; Girotto et al. 2007; Mercier et al. 2017; Stragà et al. 2022), and not necessarily when reflecting on the actual and counterfactual past.

Broadly, studies suggest that children typically begin to think counterfactually about past events around 6 years of age (Beck 2020; Gautam et al. 2019; Kominsky et al. 2021; McCormack et al. 2019, 2020; Rafetseder et al. 2013; Redshaw and Suddendorf 2020; but see Nyhout and Ganea 2019). It remains unclear, however, how controllability influences children's counterfactual thinking. In one study, Nyhout and Ganea (2020) presented 3.5‐ to 8‐year‐olds with vignettes about an event with two causes: one controllable cause and one uncontrollable cause. In one of these vignettes, a boy named Andy was doing some drawing on his front porch, but he went inside to get some juice (controllable) and the wind blew his drawings away (uncontrollable). Children were asked why the outcome occurred (“Why are Andy's drawings gone?”) and how it could have been prevented (“What should have happened so that Andy's drawings would not be gone?”). As has been found in adults (Girotto et al. 2007; Frosch et al. 2015), older children were more likely than younger children to identify the controllable cause as key to preventing the outcome. However, because the two causes in these vignettes varied on dimensions other than controllability (e.g., the weather directly caused the outcome, whereas the character action only indirectly caused the outcome by omission), it is difficult to ascertain whether controllability per se was driving the developmental transition. Furthermore, the functional theory of counterfactual thinking would suggest that children should focus on controllable rather than uncontrollable counterfactuals after experiencing an outcome *themselves* (Maloney and Egan 2017; Roese and Epstude 2017), which was not examined in this study.

Children's experiences of the counterfactual emotions of regret and relief can provide insight into whether they tend to focus on controllable or uncontrollable alternatives to personal past events. In a typical counterfactual emotion task, children are presented with two boxes—one concealing a large prize and one concealing a small prize—and are asked to open one box (Amsel and Smalley 2000). After making their selection and rating their emotion toward the contents of the chosen box, children are then shown the contents of the alternative box. Such studies usually find that children begin to report a negative change in emotion (i.e., regret) after seeing a better alternative prize around age 5 to 6 and a positive change in emotion (i.e., relief) after seeing a worse alternative outcome around age 7 to 8 (O'Connor et al. 2015; O'Connor et al. 2012; Van Duijvenvoorde et al. 2014; Weisberg and Beck 2010, 2012). In a different paradigm, children opted to spin one of two “wheels of fortune” that led to alternative outcomes (Guerini et al. 2020). Critically, children were either shown only the outcome of their chosen wheel or outcomes of both their chosen wheel and the unchosen wheel. Consistent with the broad pattern of findings from studies using the box paradigm, children from around age 6 reported a stronger negative emotion (i.e., regret) when the outcome of both wheels was revealed than when only their selected wheel was revealed.

In one particularly relevant study of children's counterfactual emotions, Weisberg and Beck (2012) manipulated the controllability of a positive or negative outcome. One group of children had complete control over their choice between two cards that concealed a better or worse outcome, a second group of children had to roll a die to determine the selected card, and a third group of children watched the experimenter roll a die to determine the selected card. The results suggested that the more control children had over the selected card, the more likely they were to experience a negative or positive change in emotion (i.e., regret or relief) after seeing the alternative card. Similarly, two recent studies have found that children were more likely to experience counterfactual emotions when given a choice between two boxes than when compelled to pick one box because they did not have the means to open the other box (Gautam et al. 2022; Jones et al. 2024). Overall, such studies indicate that the controllability of an outcome is associated with the strength of children's counterfactual emotions, potentially suggesting that children tend to focus on controllable counterfactuals from the time they begin to experience regret and relief. In all of these studies, however, the experimental procedure involved only one direct causal factor in each child's outcome (i.e., one card/box choice or one die roll), and so it is difficult to determine whether children preferentially focus on controllable counterfactuals in scenarios where controllable and uncontrollable factors have made equivalent contributions to the outcome.

In a final relevant study, Stragà et al. (2022) presented 8‐ to 12‐year‐olds and adults with a time‐limited task in which they had to sequentially connect numbers and letters on a sheet of paper, alternating between numbers and letters. After completing the task, children were instructed to evaluate their performance by finishing the following thought: “The game would have been better for me if…”. Results suggested that children were significantly more likely to generate a statement with a controllable counterfactual than an uncontrollable counterfactual, and that children were more likely to generate controllable counterfactuals than adults. One possible reason for this effect is that, by adulthood, people already have a wealth of experience in learning from actual and counterfactual‐past choices, such that they might no longer need to focus on specific controllable counterfactuals in order to make better future decisions. Children, by contrast, are still learning how to optimize their decision‐making across many contexts (Gautam and McAuliffe 2024; O'Connor et al. 2014) and thus may have more to gain from considering controllable counterfactuals than adults. Because the controllable and uncontrollable counterfactuals in Stragà et al. (2022) study were self‐generated by the children, however, they were not necessarily equivalent on dimensions other than controllability. Furthermore, given that children begin to think counterfactually about past events around 6 years of age, it is critical to examine younger samples to elucidate the initial emergence of controllability effects on such thinking.

## 1 The Current Studies

Across two preregistered studies, we investigated the developmental origins and trajectory of children's tendency to focus on controllable rather than uncontrollable counterfactuals. In Study 1, we investigated if children experienced stronger emotions toward a cause that was within their control compared to a cause that was out of their control, where both causes involved counterfactuals that would have equally changed a positive or negative outcome. In Study 2, we examined whether children experienced stronger emotions toward a negative outcome that was foreseeable than an equivalent outcome that was unforeseeable, where only the foreseeable outcome involved a counterfactual that children could have apparently known in advance and controlled.

## 2 Study 1

In the first study, we implemented a novel paradigm in which there were two direct causes of a positive or negative outcome. The two causes were essentially equivalent, except that one cause was controlled by the child and one cause was out of the control of the child, which allowed us to directly examine whether children experienced stronger emotions toward the cause with a controllable counterfactual or the cause with an uncontrollable counterfactual. Children aged 4–9 years were initially given four stickers and presented with two pairs of boxes, with each pair of boxes concealing one white ball and one black ball. Children selected a box from one pair (i.e., the controllable selection) and the experimenter spun a wheel to select a box from the other pair (i.e., the uncontrollable selection). Both selected boxes were then opened by the experimenter simultaneously, and children were assigned to either the *winning* condition (gain four extra stickers) if the boxes concealed matching balls or the *losing* condition (lose all four stickers) if the boxes concealed nonmatching balls. Finally, children were asked to report and compare their emotional experiences toward each selected box.

If children tend to preferentially focus on counterfactuals that were within their control, then they should report stronger emotions toward the box that was selected with their control than the box that was selected without their control. Specifically, children in the losing condition should report feeling *worse* toward the controllable box than the uncontrollable box, and children in the winning condition should report feeling *better* toward the controllable box than the uncontrollable box.

### 2.1 Method

#### 2.1.1 Participants

As preregistered (https://osf.io/u7rjn/?view_only), 192 children (96 males and 96 females) aged between 4.01 and 9.96 years (M = 7.03 years, SD = 1.68 years) were included in the analyses, with 32 children each aged 4, 5, 6, 7, 8, and 9 years. An additional five children were excluded due to experimenter error (*n* = 1), failure of manipulation checks (*n* = 2), and withdrawing from the study (*n* = 2). Children were either allocated to the *winning* condition (*n* = 96, 48 males, 48 females, M = 7.05 years, SD = 1.69 years, range = 4.01–9.96 years) or *losing* condition (*n* = 96, 48 males, 48 females, M = 7.01 years, SD = 1.67 years, range = 4.20–9.96 years), depending on their performance on the task. Once 16 children of a given age had been allocated to each condition, any additional children of that age were excluded (before rating their emotions) if they ended up falling into that condition. A post hoc power analysis revealed that we had a 92% chance of detecting a medium‐sized age effect (*r* = 0.30) for the critical comparison questions in each condition (*n* = 96 per condition). The sample mostly consisted of White and middle‐class children from an Australian city. Children were tested at a local museum (*n* = 183) or in a university lab space (*n* = 9). Data were collected from December 2021 to March 2022.

#### 2.1.2 Procedure

**FIGURE 1:**
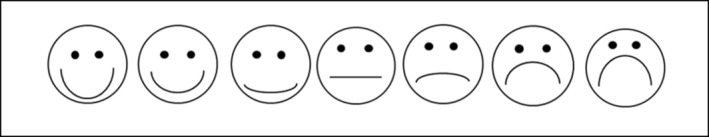
Post‐Outcome Emotion Rating Scale. Scale of faces (from Gautam et al. 2017) children used to report their post‐outcome emotion ratings for each selected box. The scale was explained to children from left to right: “This face is extremely happy, this face is very happy, this face is a little bit happy, this face is not happy or sad, this face is a little bit sad, this face is very sad, and this face is extremely sad.”

Children were first introduced to a 7‐point Likert scale of emotional faces (ranging from extremely happy to extremely sad; see Figure 1 for a depiction and scale description). Before the main task, children were given four stickers and presented with two pairs of boxes (one pair teal and one pair orange; refer to Supporting Information Figure S1 for images of the boxes). The experimenter explained (and physically demonstrated) that each box within a pair would conceal either a black or white ball, and the position of each ball would be unknown to children. Children were then told that one box from each pair would be selected, and that if the selected boxes contained two matching‐colored balls, they would win four more stickers (these children were allocated to the *winning* condition). They were also told, however, that if the selected boxes contained two nonmatching‐colored balls, then they would lose all four of their stickers (these children were allocated to the *losing* condition). A memory check was then conducted to assess children's understanding of how the different ball combinations resulted in a gain or loss of stickers, such that children had to repeat that matching balls led to a win and nonmatching balls led to a loss before moving on. Next, the experimenter explained that children would select a box from one pair (a controllable selection), but the experimenter would spin a wheel that would select a box from the other pair (an uncontrollable selection). After both selections had been made, the experimenter revealed the contents of the two selected boxes by lifting each lid simultaneously.

**FIGURE 2:**
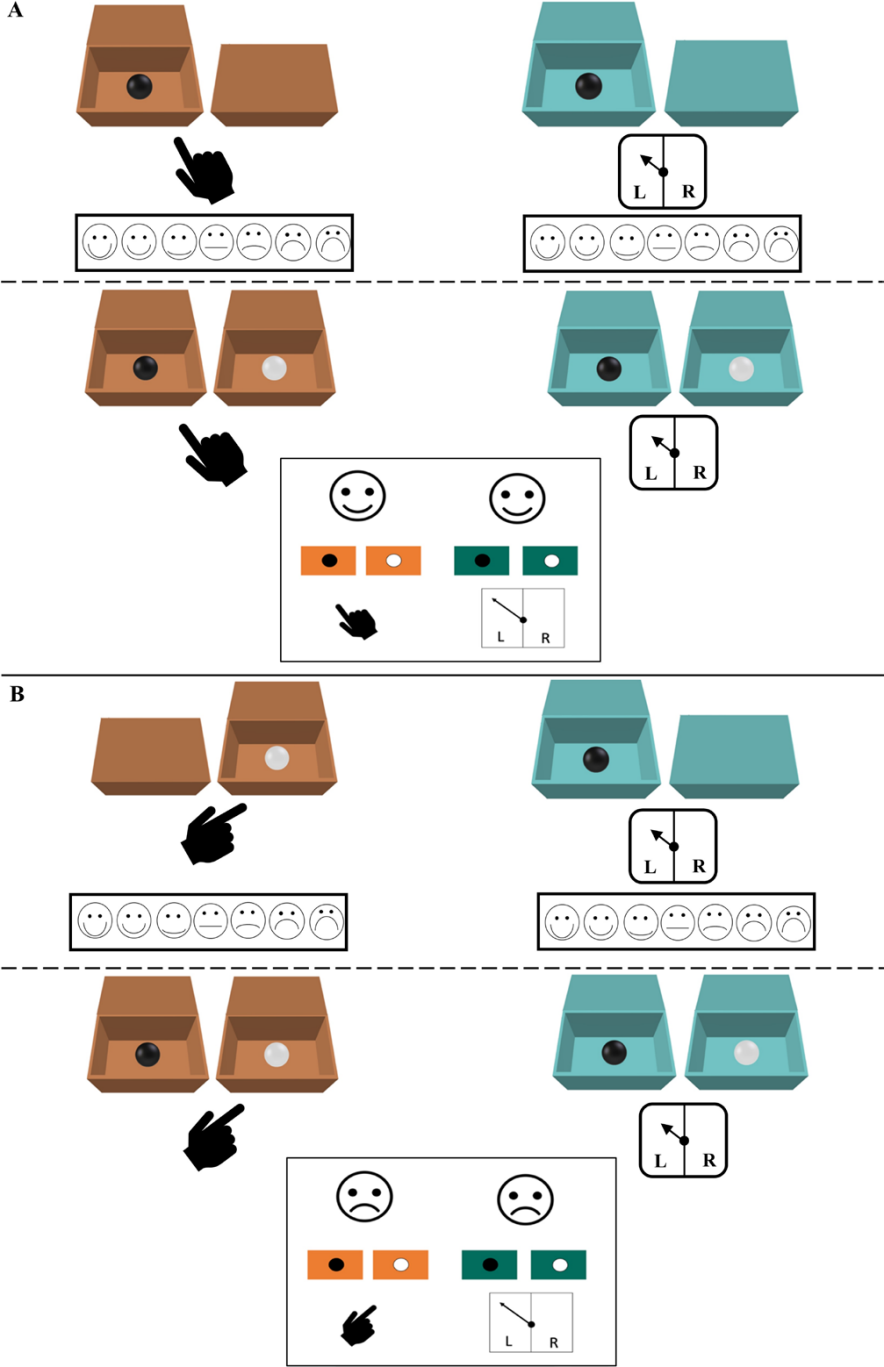
Depictions of the measures presented to children after box selection. (A) A depiction of the winning condition, showing one possible configuration where the child‐ and wheel‐selected boxes had matching balls. Children were first asked to report emotion ratings about each selected box (top panel) and then asked to rate which selected box they felt better about using a stimulus sheet as a guide (bottom panel). (B) A depiction of the losing condition, showing one possible configuration where the child‐ and wheel‐selected boxes had nonmatching balls. Children were first asked to report emotion ratings about each selected box (top panel) and then asked to rate which selected box they felt worse about using a stimulus sheet as a guide (bottom panel).

After finding out that they had won or lost the game, children first reported their post‐outcome emotion ratings toward each selected box (child‐selected and wheel‐selected) on the 7‐point Likert scale of emotional faces (see Figure 2 for a depiction of the procedure). Children were then prompted to think about the counterfactual outcomes that would have occurred had each selected box been different (i.e., “What would have happened if you selected this box instead?” and “What would have happened if the wheel landed on this box instead?”). Critically, as shown in Figure 2, children were next asked a comparison question: in the winning condition, they were asked which selected box they felt *better* about (child‐selected or wheel‐selected box) and in the losing condition, they were asked which selected box they felt *worse* about (child‐selected or wheel‐selected box). Children were also asked to justify why they felt better (in the winning condition) or worse (in the losing condition) about that particular box. Regardless of condition, all children received eight stickers at the end of the study.

#### 2.1.3 Counterbalancing

We counterbalanced (1) the colors of the boxes that children and the wheel were selecting from (i.e., child selecting from teal boxes and wheel selecting from orange boxes, or vice versa), (2) the position of the balls inside of the boxes (i.e., they were ordered black–white or white–black in the teal boxes, and black–white or white–black in the orange boxes), (3) the order that the boxes were selected (i.e., the child selected a box first and the wheel second, or vice versa), and (4) the order in which the experimenter asked how the child felt about each selected box, such that the experimenter would ask about the child‐selected box or the wheel‐selected box first.

### 2.2 Results

#### 2.2.1 Post‐Outcome Emotion Ratings

Our preregistered analyses of children's post‐outcome emotion ratings (range from −3 = extremely sad to 3 = extremely happy) included a mixed ANOVA, with a within‐subject factor of selector (child vs. wheel), a between‐subject factor of outcome (positive vs. negative), and a between‐subject factor of age (continuous and mean centered). After conducting the study, however, we discovered that a previous study had found effects of temporal order on children's counterfactual thinking about outcomes with two sequential causes (Meehan and Byrne 2005), and another study had found that 9‐ and 11‐year‐olds exhibited a temporal order bias when making judgments about others' regret (Payir and Guttentag 2019). We therefore included an additional between‐subject factor of selection order (child first vs. wheel first). This analysis revealed a significant main effect of outcome, *F*(1, 187) = 171.00, *p* < 0.001, *η*_*p*_^*2*^ = 0.48, with winning children unsurprisingly reporting higher mean levels of happiness toward both selections (M = 2.29, SD = 0.99) than losing children (M = 0.36, SD = 1.62). All other effects, including those involving age, were nonsignificant, all *F* < 5.16, *p* > 0.056, *η*_*p*_^*2*^ < 0.02, with the exception of the three‐way selector × outcome × selection order effect, *F*(1, 187) = 4.30, *p* = 0.039, *η*_*p*_^*2*^ = 0.02 (see Supporting Information Figures S2 and S3 for full descriptive data).

**FIGURE 3:**
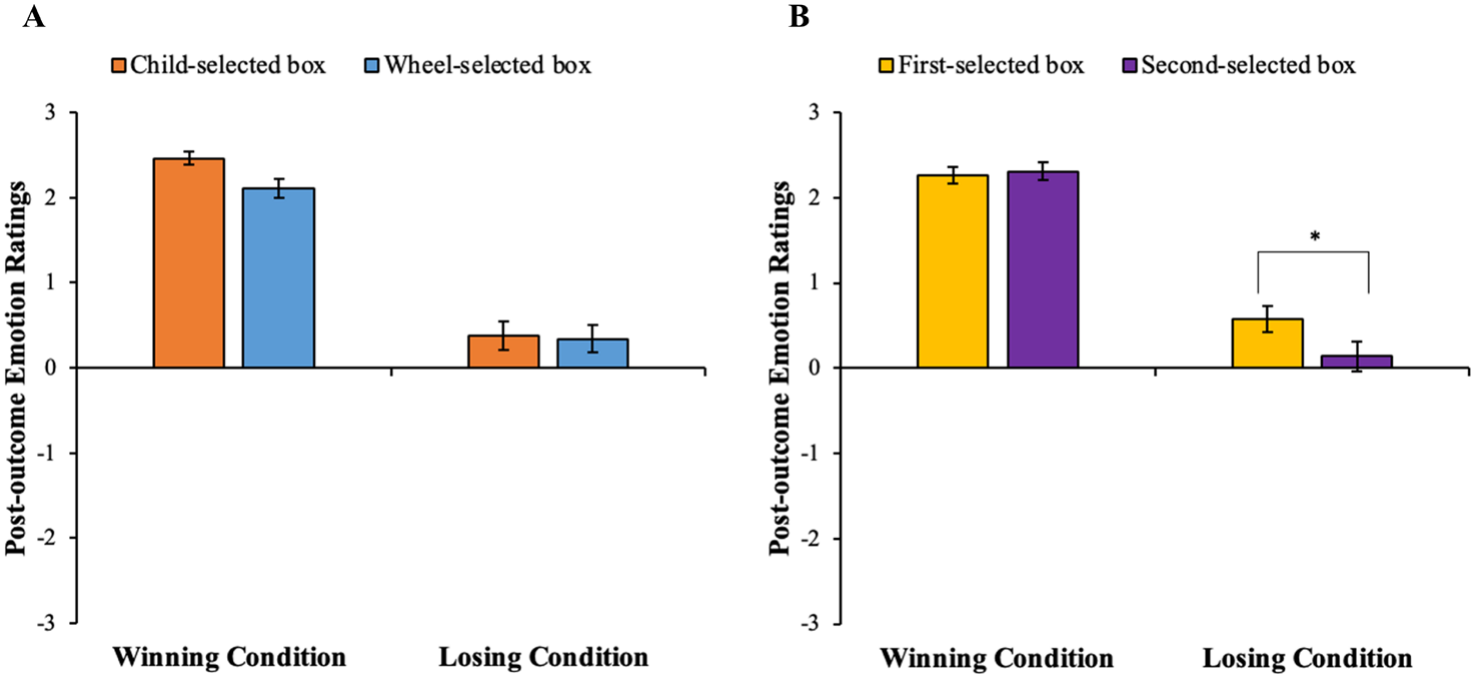
Post‐outcome emotion ratings as a function of box selector and choice order. (A) Children's post‐outcome emotion ratings for each selected box (raw scores), split by condition and whether the box was selected by the child (orange bars) or the wheel (blue bars). (B) The same emotion ratings for each selected box (raw scores), split by condition and whether the box was selected first (yellow bars) or second (purple bars), regardless of whether it was selected by the child or wheel. Error bars represent standard errors. *p < 0.01.

To make this complex three‐way interaction more interpretable, we first ran an equivalent univariate ANOVA in which we collapsed the within‐subject selector factor into a difference score (i.e., the rating for child‐selected box *minus* the rating for wheel‐selected box), which resulted in an equivalent significant two‐way interaction: outcome × selection order, *F*(1, 187) = 4.30, *p* = 0.039, *η*_*p*_^*2*^ = 0.02 (see Figure 3). We then used a Bonferroni correction for follow‐up pairwise comparisons (resulting in an adjusted alpha of 0.025), which revealed that, among winning children, the difference scores did not significantly vary as a function of selection order, *F* (1, 187) = 0.08, *p* = 0.784, *η*_*p*_^*2*^ < 0.01 (child first, M = 0.30, SE = 0.24; wheel first, M = 0.39, SE = 0.24). Among losing children, however, the difference scores did significantly vary as a function of selection order, *F* (1, 187) = 7.07, *p* = 0.009, *η*_*p*_^*2*^ = 0.04 (child first, M = 0.48, SE = 0.24; wheel first, M = −0.43, SE = 0.24). Because of the way the difference scores were calculated, this indicates that losing children felt significantly sadder about the second selected box than about the first selected box.

#### 2.2.2 Comparison Question

As preregistered, children's responses to the comparison question were analyzed separately for those who won and those who lost. This was due to the difference in dependent variables across children (i.e., winning children were asked which box they felt “better” about and losing children were asked which box they felt “worse” about).

##### 2.2.2.1 Winning Condition

As children could select feeling better about the child‐selected box or the wheel‐selected box after seeing inside the alternative boxes, we first examined if their responses significantly differed from an a priori chance level of 50% (as preregistered). A binomial test revealed that, across ages, children in the winning condition reported feeling better about the child‐choice box significantly more often (63.5%) than chance, *p = *0.010. A preregistered point–biserial correlation, however, found no significant relationship between age and children's responses, *r*(94) = 0.02, *p* = 0.863.

**FIGURE 4:**
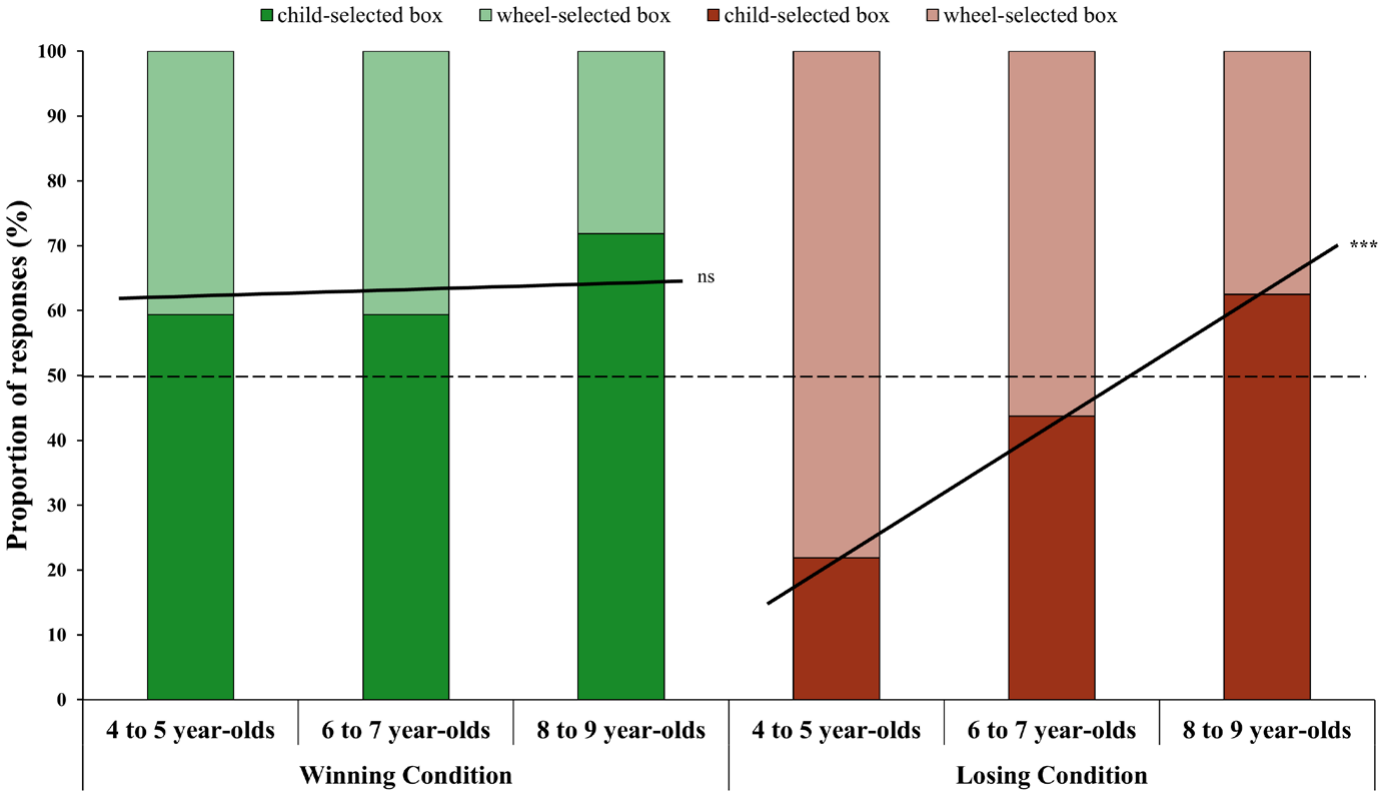
Comparison question results for the winning and losing conditions split by age. Children in the winning condition who reported feeling better about the child‐selected box (dark green bars) and the wheel‐selected box (light green bars). Children in the losing condition who reported feeling worse about the child‐selected box (dark red bars) and the wheel‐selected box (light red bars). ***p < 0.001 (continuous age effect).

An exploratory logistic regression examined whether children's responses to the comparison question (0 = better toward the wheel‐selected box, 1 = better toward the child‐selected box) varied as a function of age and selection order (1 = child selected a box first, 2 = wheel selected a box first). In Step 1, the regression constant was significant, *b* = −0.56, SE = 0.21, Wald *χ* ^*2*^ (1, *N* = 96) = 6.86, *p* = 0.009, *w* = 0.27, confirming that children reported feeling better about the child‐selected box than the wheel‐selected box (see Figure 4). Age did not predict children's responses, *b =* −0.02, SE = 0.13, Wald *χ* ^*2*^ (1, *N* = 96) = 0.03, *p* = 0.862, *w* = 0.02 and nor did selection order of boxes, *b* = −0.83, SE = 0.44, Wald *χ* ^*2*^ (1, *N* = 96) = 3.60, *p* = 0.058, *w* = 0.19. In Step 2, there was also no evidence of an age × selection order interaction, *b* = −0.27, SE = 0.26, Wald *χ* ^*2*^ (1, *N* = 96) = 1.04, *p* = 0.308, *w* = 0.10.

##### 2.2.2.2 Losing Condition

A binomial test revealed that, across ages, children in the losing condition reported feeling worse about the child‐selected box at a level (42.7%) no different from chance, *p = *0.184. Critically, however, children's responses significantly varied with age, *r*(94) = 0.36, *p* < 0.001. On the whole, this finding suggests that, with increasing age, children were more likely to feel stronger toward the box for which the selection was within their control. Post hoc binomial tests (50% chance level; adjusted α = 0.017) revealed that 4‐ to 5‐year‐olds chose the child‐selected box significantly less often than chance (21.9%, *p* = 0.002), whereas 6‐ to 7‐year‐olds (43.8%, *p* = 0.600) and 8‐ to 9‐year‐olds (62.5%, *p* = 0.215) did not differ from chance (see Figure 4).

An exploratory logistic regression examined whether children's responses (0 = worse toward the wheel‐selected box, 1 = worse toward the child‐selected box) to the comparison question varied as a function of age and selection order (1 = child selected a box first, 2 = wheel selected a box first). In Step 1, the regression constant was nonsignificant, *b* = 0.29, SE = 0.21, *Wald χ* ^*2*^ (1, *N* = 96) = 2.03, *p* = 0.155, *w* = 0.15, confirming that, on average, children did not feel worse about the child‐selected box than the wheel‐selected box. Again, however, age did significantly predict children's responses, *b* = 0.48, SE = 0.14, *Wald χ* ^*2*^ (1, *N* = 96) = 11.50, *p* < 0.001, *w* = 0.35, suggesting that children were more likely to report feeling worse about the child‐selected box with increasing age (see Figure 4). Further, order of box choice did not predict children's responses, *b* = 0.55, SE = 0.45, *Wald χ* ^*2*^ (1, *N* = 96) = 1.48, *p* = 0.223, *w* = 0.12. In Step 2, there was no evidence of an age × selection order interaction, *b* = 0.14, SE = 0.29, *Wald χ* ^*2*^ (1, *N* = 96) = 0.23, *p* = 0.632, *w* = 0.04.

#### 2.2.3 Verbal Responses

Children's verbal responses were coded for whether they referred to a counterfactual in their justification following the comparison question. An example of a response with a counterfactual aspect in the winning condition is “because the wheel could have chosen any box but I chose the right box,” and in the losing condition “I could have chosen the other box and that means I would have won”. An example of a response without a counterfactual aspect in the winning condition is “because I won” and in the losing condition is “because I didn't win.” Further, we coded if children's responses referred to the controllability of either selection (regardless of whether they also referred to a counterfactual). An example of a child's response referring to their own selection being within their control is “the box I chose was in my control” and an example of a response referring to the wheel's selection being out of their control is “it was lucky the wheel chose the box that won.” Forty‐eight transcribed responses (25% of total participants, eight responses from each age and 24 responses from each condition) were also coded by a trained research assistant. There was almost perfect agreement (see McHugh 2012) between raters for coding of both counterfactual aspects, *κ* = 0.95, *p* < 0.001, and controllability aspects in children's responses, *κ* = 0.81, *p* < 0.001.

A preliminary analysis revealed that children were significantly more likely to give no substantive verbal response (*n* = 54) with decreasing age, *r*(190) = −0.17, *p =* 0.020, and these children were therefore removed from subsequent correlational analyses to avoid confounding age with verbosity. Among the 138 children who provided a substantive response, point–biserial correlations revealed that the likelihood of referring to a counterfactual (30.4%; *n* = 42/138) significantly increased with age, *r*(136) = 0.21, *p* = 0.013, and the likelihood of referring to the controllability of the box selections (29.7%; *n* = 41/138) also significantly increased with age, *r*(136) = 0.28, *p* < 0.001 (refer to Table S1 in the Supporting Information for full verbal response information and further analyses of children's responses).

### 2.3 Discussion

In Study 1, children were provided with an opportunity to directly compare two past events that had equally caused a positive or negative outcome, where one event involved a controllable counterfactual and one event did not. For the positive outcome, children's post‐outcome emotion ratings did not significantly vary between the controllable and uncontrollable boxes. Consistent with previous research (McCormack et al. 2016), however, the comparison question was more sensitive to differences in children's emotions, with participants reporting they felt significantly better toward the controllable box selection than the uncontrollable box selection. One interpretation is that this result reflects children's increased counterfactual feelings of relief toward the controllable selection. That is, consistent with the functional theory of counterfactual thinking (Epstude and Roese 2008), children may have felt better toward the controllable selection because it involved a counterfactual that was beneficial to consider for improving future behavior (i.e., a box selection that children should avoid in the future). Yet, another interpretation is that these children did not typically consider how to improve their behavior at all, given that they had just achieved the optimal outcome. Indeed, many of these children's reports may have simply reflected their general self‐centeredness, in that they preferred to attribute the positive outcome to their own action rather than any other action, regardless of the counterfactuals involved. This interpretation would explain the lack of an age effect in the positive condition, which does not align with the well‐documented changes in counterfactual thinking between 4 and 9 years of age (Gautam et al. 2019; McCormack et al. 2019, 2020; Rafetseder et al. 2013; Redshaw and Suddendorf 2020).

When the outcome was negative, children's post‐outcome emotion ratings also did not significantly vary between the controllable and uncontrollable box selections. Rather, these ratings suggested that children felt sadder toward the box that was selected second than the box that was selected first, regardless of the selector. One possibility is that children attributed greater causality to the most recent box selection (see Meehan and Byrne 2005; Payir and Guttentag 2019), but this interpretation should be taken with caution given that the effect was not found for the critical comparison question. Instead, the responses to the comparison question revealed that, with increasing age, children showed a significant reduction in the bias of feeling worse about the uncontrollable selection. Consistent with the functional theory of counterfactual thinking (Epstude and Roese 2008) and the broader literature on the development of counterfactual thinking (Gautam et al. 2019; McCormack et al. 2019, 2020; Rafetseder et al. 2013; Redshaw and Suddendorf 2020), this finding might suggest that older children are more likely than younger children to experience regret when reflecting on negative past outcomes involving controllable counterfactuals. Nonetheless, even the 8‐ to 9‐year‐old children were still not significantly more likely than the chance to report feeling worse about the child‐selected box than the wheel‐selected box. As for the positive condition, it is possible that younger children were responding with a self‐centered bias in the negative condition—such that they simply preferred to attribute the outcome to any action but their own—but with the emergence of counterfactual thinking, children gradually shifted away from this bias to increasingly focus on the more controllable selection.

Beyond the issues with interpreting the significant and nonsignificant findings, there was an important limitation with how controllability was manipulated in the first study. That is, even though children possessed control over the box they *selected*, they arguably had no genuine control over the eventual *outcome*, as they could not have possibly foreseen what was inside each box before the contents were revealed. In other words, there was no legitimate reason for children to think “I should have known better” after finding out they had lost. Furthermore, assuming that the contents of the boxes would be randomized each time, there is also no way that children could have learned to make a better selection in the future. Therefore, the link between the results of this first study and the functional theory of counterfactual thinking is only indirect.

## 3 Study 2

When people perceive counterfactuals to have greater controllability, it is often because the actual and counterfactual outcomes of a past choice could have been foreseen. In many legal systems, for instance, the judgment that a defendant should have foreseen—but did not foresee—the negative outcome of a past action is a key element separating negligence from innocence (Green 1961; Owen 2007). More broadly, foreseeability may be a critical feature by which people determine the controllability of counterfactuals, thus making foreseeability an ideal factor to manipulate in tests of the functional theory of counterfactual thinking. Although the perceived foreseeability of past events does indeed influence counterfactual thinking in adults (Markman and Tetlock 2000), it remains unclear whether children likewise account for foreseeability when thinking counterfactually. In one relevant developmental study, McCormack et al. (2016) manipulated the degree of risk in a box selection task, such that 6‐ to 9‐year‐olds could either choose a box that would lead to 7 or 10 points (low risk) or a box that would lead to 1 or 16 points (high risk). One interpretation of this set‐up is that the outcome of the low‐risk box was less variable and therefore more foreseeable than the outcome of the high‐risk box. Nonetheless, the results showed that children's likelihood of experiencing regret and relief did not vary with risk taking, suggesting that the degree of foreseeability did not impact their counterfactual emotions. A more direct test of the role of foreseeability in children's counterfactual emotions, however, would compare their emotional experiences following choices where the actual and counterfactual outcomes could have and could not have been precisely foreseen.

Because the lack of foreseeability constrained interpretations of our findings in Study 1, in Study 2 we examined if 4‐ to 9‐year‐old children felt stronger regret toward a selection for which they apparently could have foreseen a negative outcome compared to a selection for which they could not have foreseen an identical negative outcome. Children were presented with two pairs of boxes with stickers concealed inside. They were instructed to select a box from each pair, and before making their selections, they were given time to handle the boxes in any manner they wished (except for opening the lids and looking inside). Critically, one pair of boxes had windows on the bottom, such that if the children had looked through these windows, they could have seen the number of stickers inside and thus apparently foreseen the actual and counterfactual outcomes of their choice. Unbeknownst to children, however, the contents of the boxes were in fact surreptitiously manipulated, such that both chosen boxes (foreseeable and unforeseeable) always contained one sticker and both unchosen boxes (foreseeable and unforeseeable) always contained five stickers—with the purpose of prompting feelings of regret toward each box choice. Finally, after seeing the contents of all these boxes and finding out about the windows, children were asked about their sadness toward the two chosen boxes.

In line with the functional theory of counterfactual thinking, it would benefit children to experience stronger feelings of sadness toward the boxes with the foreseeable outcomes, as focusing on these outcomes should enable them to make more adaptive decisions when confronted with similar choices with foreseeable outcomes in the future. We did not implement a positive condition in Study 2, given that children may not have any reason to think of how they could have foreseen a positive outcome after experiencing such an outcome.

### 3.1 Method

#### 3.1.1 Participants

As preregistered (https://osf.io/q748n/?view_only), 144 children (72 males and 72 females) aged between 4.01 and 9.91 years (M = 6.96 years, SD = 1.70 years) were included in the analyses, with 24 children each aged 4, 5, 6, 7, 8, and 9 years. An additional three children were excluded due to an existing clinical diagnosis (*n* = 2) and insufficient proficiency in English (*n* = 1). A post hoc power analysis revealed that we had a 98% chance of detecting a medium‐sized age effect (*r* = 0.30) for the critical comparison question (*N* = 144). The sample consisted of mostly White and middle‐class children from an Australian city. Children were tested at a local museum (*n* = 89) or in a university lab space (*n* = 58). Data were collected from June 2022 to September 2022.

#### 3.1.2 Procedure

**FIGURE 5:**
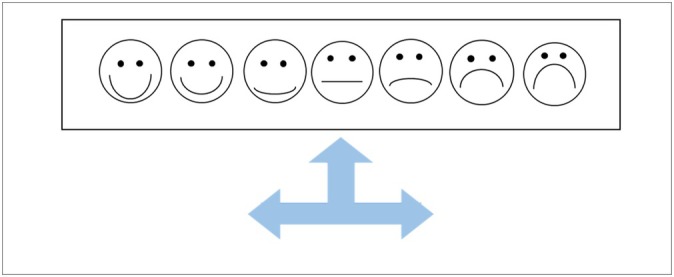
Three‐Pronged Arrow Scale presented in Study 2. Three‐pronged arrow used for emotion change ratings, explained to children as (from left to right): “These are arrows that go on top of any of the faces like this. Now wherever these arrows are placed, each arrow always means the same thing. This arrow means happier; this is because it is always pointing toward the happy faces. This arrow means the same as the face that was pointed to. This arrow means sadder; this is because it is always pointing toward the sad faces.”

Children were again introduced to the 7‐point Likert scale of emotional faces (as in Study 1), but this time it was accompanied by a three‐pronged arrow as in Weisberg and Beck's (2012) study (representing relative emotions of *happier*, *sadder*, or *the same*). After the experimenter explained the arrows to children (see Figure 5), the three‐pronged arrow was placed on the neutral face and children were asked to report what each arrow represented. This process was then repeated for the end faces of the scale (i.e., after the three‐pronged arrow was placed on the extremely happy face and the extremely sad face). This training check was implemented to demonstrate to children that they could report feeling *happier*, *sadder*, or *the same* even if their initial emotion rating was at the end of the scale.

**FIGURE 6:**
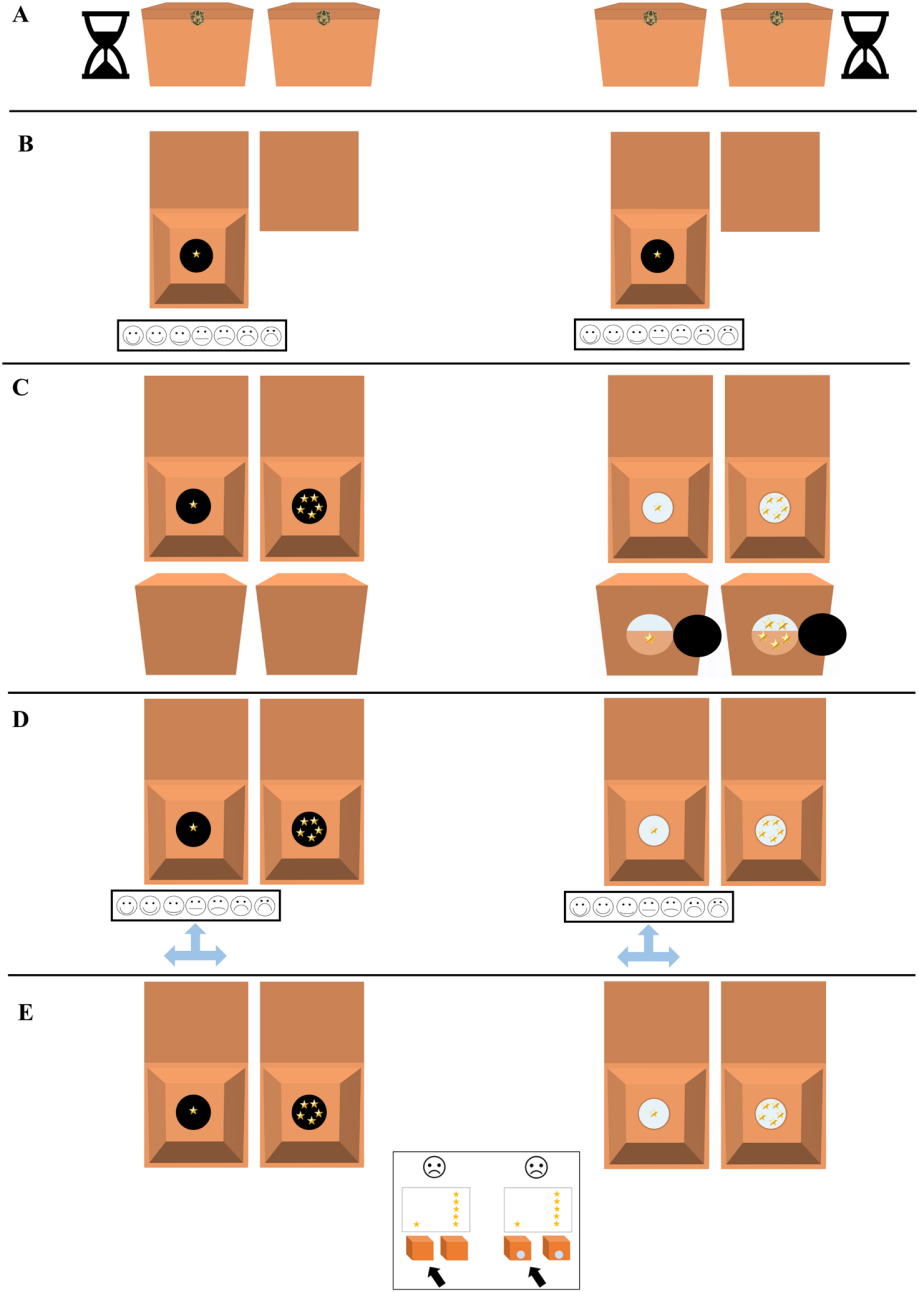
A depiction of the Study 2 procedure. (A) Children were given 15 s to manipulate one pair of boxes in any manner and then chose one box out of that pair, and this process was then repeated for the other pair of boxes. (B) Both chosen boxes were first revealed to have one sticker each, and children were asked to rate their emotion toward each selected box. (C) Both nonchosen boxes were then revealed to have five stickers each; before one pair of boxes was revealed to have windows on the bottom. The experimenter demonstrated that if you picked up these boxes and looked through the windows, you could see the number of stickers inside and also demonstrated that this was not the case for the other pair of boxes. (D) Children were then asked to report an emotion change rating toward each selected box using the three‐pronged arrow. (E) Finally, children were shown a stimulus sheet to assist with the comparison question about which selected box they felt sadder toward. For all children, the stimulus sheet showed that the chosen foreseeable box and the chosen unforeseeable box both had one sticker, whereas the nonchosen boxes both had five stickers (where the arrangement of the boxes in the stimulus sheet shown to each child mapped onto the child's actual selections).

The experimenter presented two pairs of closed orange boxes and explained to children that they would be selecting one box from each pair to search for stickers (refer to Supporting Information Figure S4 for images of the boxes). Prior to each choice, however, children were given 15 seconds in which they could pick up and manipulate the boxes in any manner except for opening the top lids. After children made their two selections, the experimenter revealed that each of the two chosen boxes contained one sticker, and children reported an initial emotion rating about each chosen box on the 7‐point Likert scale of faces (see Figure 5). Next, the contents of the nonchosen boxes were revealed to contain five stickers (as surreptitiously ensured by the experimenter). Critically, the experimenter then demonstrated that one pair of boxes had windows on the bottom, such that children apparently could have foreseen the outcome of their choice if only they had looked underneath the boxes. The experimenter also demonstrated, by contrast, that the other pair of boxes had no such windows. Children then reported their emotion change rating for each selected box with the three‐pronged arrow (i.e., “Do you feel happier, sadder or the same about this box you chose?”; see Figure 6).

Importantly, the experimenter next asked children a comparison question regarding whether they felt worse about the chosen box with a window or the chosen box without a window, and children were also prompted to provide a verbal justification for their response (i.e., “Why do you feel worse about this box?”; see Figure 6). Finally, children were asked two manipulation checks: first to check if they understood they could use the windows on the foreseeable boxes to prevent the negative outcome (i.e., “If we were to play the game again what would you do in the 15 seconds before choosing your box for the boxes with windows?” and “What would you do in the 15 seconds before choosing your box for the boxes with no windows?”), and second to check that children actually wanted to receive more stickers (i.e., “If you could choose your boxes again which boxes would you want?”). All children indicated that they would choose the boxes with more stickers, and all children received 12 stickers as compensation at the end of the task.

#### 3.1.3 Counterbalancing

We counterbalanced (1) the position of the boxes, such that the pair of boxes with windows were either on the left or right and (2) the order in which children selected the boxes, such that they either first selected from the boxes with windows or the boxes without windows. The measures for each selected box were administered in the same order in which children had selected them.

### 3.2 Results

#### 3.2.1 Initial Emotion Ratings

There was no significant difference between children's initial emotion ratings on the 7‐point scale (range from −3 = extremely sad to 3 = extremely happy) toward each chosen box (foreseeable, M = 1.63, SD = 1.48, and unforeseeable, M = 1.55, SD = 1.48), *F*(1, 142) = 0.38, *p* = 0.541. This finding indicates that children were generally happy about receiving one sticker from each chosen box. As intended by the task design, it also indicates that children did not differentiate between the foreseeable and unforeseeable boxes before being shown that the foreseeable boxes had windows.

#### 3.2.2 Emotion Change Ratings

As children could select from three emotion change ratings (*happier*, *sadder*, or *the same*) after the contents and foreseeability of each alternative box were revealed, we first examined if their responses significantly differed from an a priori chance level of 33.3%. As preregistered, a binomial test revealed that, across ages, children reported feeling sadder significantly more often than chance after the alternative boxes were revealed for the window boxes (45.1%, *p* = 0.002) and the no‐window boxes (41%, *p* = 0.032). Point–biserial correlations were then conducted to examine whether these effects varied as a function of age. These revealed that children's likelihood of feeling sadder significantly increased with age for both window and no‐window boxes, *r*(142) = 0.40, *p* < 0.001, *r*(142) = 0.23, *p* = 0.005, respectively. Post hoc binomial tests (adjusted *α* = 0.017) revealed 4‐ to 5‐year‐olds felt sadder about the foreseeable (20.8%, *p* = 0.042) and unforeseeable (31.3%, *p* = 0.671) boxes at levels no different from chance, 6‐ to 7‐year‐olds felt sadder about the foreseeable (41.6%, *p* = 0.142) and unforeseeable (39.6%, *p* = 0.220) boxes at levels no different from chance, and 8‐ to 9‐year‐olds felt sadder about the foreseeable (72.9%, *p* < 0.001) and unforeseeable (52.1%, *p* = 0.005) boxes at levels greater than chance. Refer to Supporting Information (Table S3) for the proportions of responses that were *happier* and *the same*.

**FIGURE 7:**
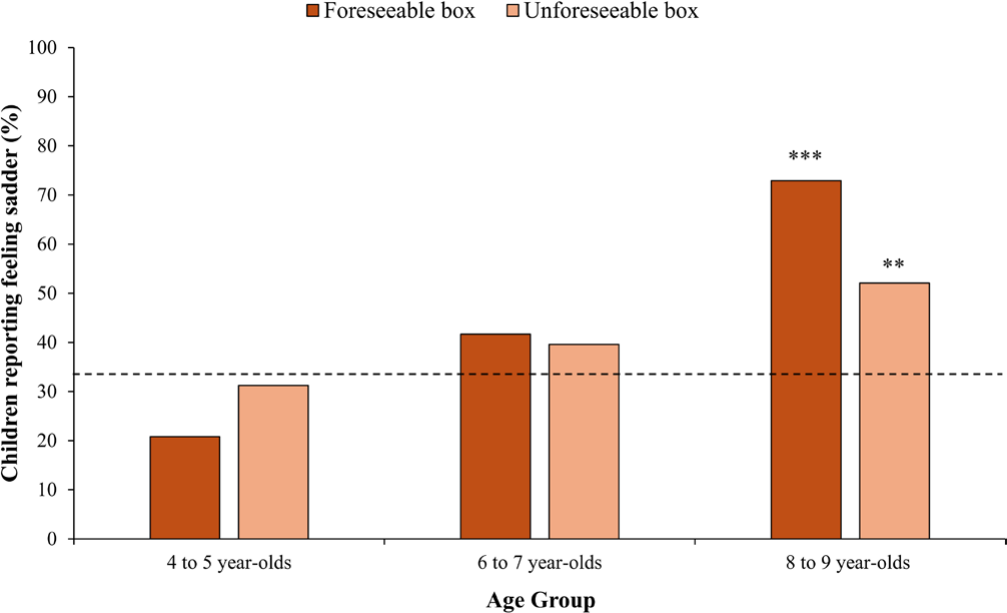
Change in emotion for the foreseeable and unforeseeable boxes. Percentage of children reporting feeling sadder about the foreseeable and unforeseeable boxes after the alternative outcomes were revealed by age groups. Asterisks indicate responses that differed significantly from chance (33.3%; adjusted α = 0.017). **p < 0.01. ***p < 0.001.

An exploratory generalized linear mixed model (with a random intercept for participant) examined whether children's changes in emotion varied with age and the foreseeability of each box. This revealed a significant main effect of age, χ^2^ (1, *N* = 144) = 17.25, *p* < 0.001, *w* = 0.35, such that with increasing age, children were more likely to report feeling sadder once the alternative outcomes were revealed across both chosen boxes. The foreseeability main effect was not significant, χ^2^ (1, *N* = 144) = 0.52, *p* = 0.469, *w* = 0.06, and neither was the foreseeability × age interaction, χ^2^ (1, *N* = 144) = 3.02, *p* = 0.085, *w* = 0.15 (see Figure 7), such that children were not more likely to report feeling sadder about the foreseeable box compared to the unforeseeable box with increasing age. When excluding children (post hoc) who failed the manipulation check about what they would do next time they played (*n* = 17; fourteen 4‐ to −5‐year‐olds and three 6‐ to 7‐year‐olds), however, the interaction was significant, χ^2^ (1, *N* = 127) = 5.13, *p* = 0.024, *w* = 0.20. Follow‐up multiple comparisons revealed that the 4‐ to 5‐year‐olds (χ^2^ (1, *N* = 34) = 3.38, *p* = 0.066, *w* = −0.32) and 6‐ to 7‐year‐olds (χ^2^ (1, *N* = 45) = 0.07, *p* = 0.791, *w* = −0.04) were more likely to report feeling sadder about the unforeseeable box than the foreseeable box (but not significantly so), whereas the 8‐ to 9‐year‐olds (χ^2^ (1, *N* = 48) = 5.84, *p* = 0.016, *w* = 0.35) were significantly more likely to report feeling sadder about the foreseeable box than the unforeseeable box (adjusted α = 0.017; see Supporting Information Figure S5).

#### 3.2.3 Comparison Question Analyses

**FIGURE 8:**
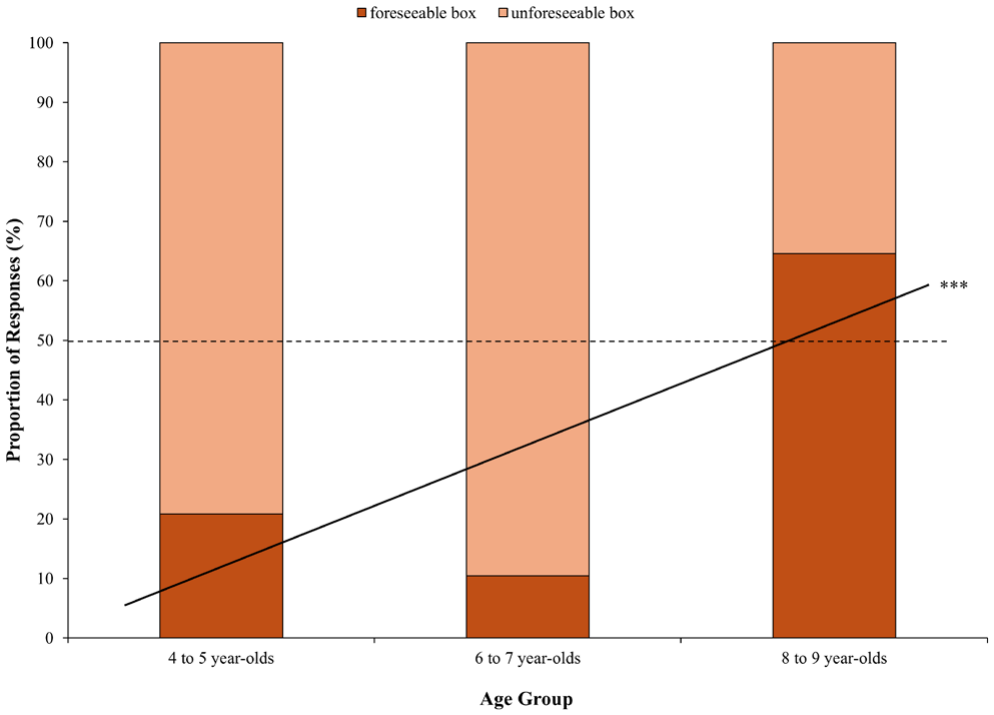
Comparison question. Proportion of children who reported feeling worse about the foreseeable box (with window) or the unforeseeable box (without window) by age group. ***p < 0.001 (continuous age effect).

As children could select feeling worse about the foreseeable box or the unforeseeable box after seeing inside the alternative boxes, we first examined if their responses significantly differed from an a priori chance level of 50%. A preregistered binomial test revealed that, across ages, only 48/144 (33.3%) of children reported feeling worse about the foreseeable box, which was significantly *less* often than chance, *p < *0.001. Critically, however, a point–biserial correlation revealed that children's responses significantly varied with age, *r*(142) = 0.37, *p* < 0.001. As shown in Figure 8, post hoc binomial tests (50% chance level) revealed that there was a large change in the percentage of children selecting the foreseeable box between the two younger age groups (15.6% on average, both *p < *0.001) and the 8‐ to 9‐year‐olds (64.5%, *p = *0.060). In summary, younger children unexpectedly possessed a bias toward feeling worse about the choice where they could *not* have foreseen the outcome, whereas older children did not possess this bias.

#### 3.2.4 Box Manipulation Analyses

Although children could have genuinely looked through the windows before selecting a box, no participants actually looked through the windows during the exploration time. Nonetheless, we conducted exploratory post hoc analyses on the effect of children's behavior during the exploration time. This involved coding whether children physically interacted with the pair of boxes during each 15 second period. Of the 123 participants who were video recorded (recordings of 21 participants were missing due to camera malfunctions or absence of consent for video recording), 81 children (65.9%) physically interacted with both pairs of boxes (4‐ to 5‐year‐olds, *n* = 16/43; 6‐ to 7‐year‐olds, *n* = 35/46; 8‐ to 9‐year‐olds, *n* = 30/34) and 42 children (34.1%) did not physically interact with either pair of boxes (4‐ to 5‐year‐olds, *n* = 27/43; 6‐ to 7‐year‐olds, *n* = 11/46; 8‐ to 9‐year‐olds, *n* = 4/34).

Point–biserial correlations revealed that the children who manipulated the boxes were more likely to report feeling worse toward the foreseeable box with increasing age, *r*(79) = 0.49, *p* < 0.001. However, for children who did not manipulate the boxes, there was no significant relationship between box choice and age, *r*(40) = −0.15, *p* = 0.342. An exploratory logistic regression then examined whether children's responses to the comparison question varied as a function of age and box manipulation (0 = no box manipulation, 1 = box manipulation). In Step 1, this analysis revealed that children who manipulated the boxes were not significantly more likely to report feeling worse toward the foreseeable box than the unforeseeable box, *b* = 0.05, SE = 0.51, *Wald χ* ^*2*^ (1, *N* = 123) = 0.01, *p* = 0.931, *w* = 0.01, although there was a significant age effect, *b* = 0.48, SE = 0.15, *Wald χ* ^*2*^ (1, *N* = 123) = 10.03, *p* = 0.002, *w* = 0.29. Critically, in Step 2, there was a significant age × box manipulation interaction, *b* = 1.23, SE = 0.39, Wald *χ* ^*2*^ (1, *N* = 123) = 9.93, *p* = 0.002, *w* = 0.28, confirming that the effect of age on selecting the foreseeable box was contingent on children having manipulated the boxes in some way (see Supporting Information for emotion change exploratory analyses).

#### 3.2.5 Verbal Responses

Children's verbal responses were coded for whether they referred to a counterfactual in their justification following the comparison question. An example of a counterfactual response from a child who reported feeling worse toward the foreseeable box is “because I definitely could have looked through the window and figured it out” and an example of a counterfactual response from a child who reported feeling worse toward the unforeseeable box is “if it had windows I could have looked in and seen how many stickers but they have no windows so I couldn't have looked in them.” An example of a response with no counterfactual aspect is “because it has no window.” Thirty‐six transcribed responses (25% of total participants, six responses from each age) were also coded by an experienced research assistant. There was perfect agreement between raters for coding of counterfactual aspects in children's responses, *κ* = 1.00, *p* < 0.001.

A preliminary analysis revealed that children were significantly more likely to give no substantive verbal response (*n* = 19) with decreasing age, *r*(142) = −0.23, *p =* 0.006. Among the 125 children who provided a response, the likelihood of referring to a counterfactual (39.2%, *n* = 49/125) significantly increased with age, *r*(123) = 0.57, *p* < 0.001. Next, an exploratory logistic regression examined whether the children who reported feeling worse toward the foreseeable chosen box were more likely to refer to a counterfactual in their justification (1 = referred to a counterfactual, 0 = did not refer to a counterfactual) when controlling for age. Among children who provided verbal responses, in Step 1, this analysis revealed that children were significantly more likely to mention a counterfactual if they reported feeling worse toward the foreseeable box (76.9% of 39 children) than the unforeseeable box (22% of 86 children), *b* = 1.89, SE = 0.52, Wald *χ* ^*2*^ (1, *N* = 125) = 13.05, *p* < 0.001, *w* = 0.32. The age effect was weaker but still significant, *b* = 0.35, SE = 0.17, *Wald χ* ^*2*^ (1, *N* = 125) = 4.15, *p* = 0.042, *w* = 0.18, and in Step 2, the age × box choice interaction was not significant, *b* = 0.39, SE = 0.35, Wald *χ* ^*2*^ (1, *N* = 125) = 1.21, *p* = 0.272, *w* = 0.10.

We further explored the contents of these counterfactual responses by coding whether the counterfactual was referring to foreseeability (e.g., “I could've checked inside to see that there was only one sticker”). For children who reported feeling worse toward the foreseeable box (*n* = 30), only one child did not refer to foreseeability within their counterfactual response but rather simply reflected on the better counterfactual outcome (i.e., “I could've had more stickers”). Similarly, for children who reported feeling worse toward the unforeseeable box (*n* = 19), only one child did not refer to foreseeability within their counterfactual response (i.e., “It would have been harder to get the 5 stickers”).

### 3.3 Discussion

Study 2 assessed whether children were more likely to experience regret when they could have foreseen the outcome before making a choice compared to when they could not have foreseen the outcome. The results suggested that, with increasing age, children were more likely to experience a negative change in emotion after the contents of the nonchosen boxes were revealed for both the foreseeable and unforeseeable boxes. Consistent with past literature (O'Connor et al. 2015; O'Connor et al. 2012; Van Duijvenvoorde et al. 2014; Weisberg and Beck 2010, 2012), this indicates that older children were in general more likely than younger children to experience a counterfactual emotion after each alternative outcome was revealed.

When children were explicitly asked to compare the selected boxes with and without windows, 4‐ to 7‐year‐old children were unexpectedly more likely than chance to feel worse about the selected box that had no window. One potential interpretation of this finding is that children of this age were more likely to feel regret toward the box they chose when they could not have possibly foreseen the outcome of that box. This interpretation seems unlikely, however, when considering that these younger children failed to report feeling sadder toward either selection significantly more often than chance (i.e., when using the three‐pronged arrow), suggesting that most of these children may not have experienced regret about their box selections at all. It is more likely, perhaps, that younger children's tendency to report feeling worse toward the unforeseeable box may have reflected their negative feelings toward the general utility of that box, without respect to its counterfactual‐past utility per se. That is, children may have simply felt frustrated or helpless toward the box that had no mechanism by which to view the concealed contents. This possibility is supported by the fact that children who reported feeling worse toward the unforeseeable box were much less likely to mention a counterfactual in their verbal justifications, suggesting these children may not have been reflecting on the past.

Another possible interpretation is that younger children tended to focus on the *future* utility of each box when answering the comparison question. That is, if children were thinking about which box would result in less favorable outcomes for *future* decisions, then they might feel worse about the unforeseeable boxes because the lack of windows would preclude them from certainly winning stickers in the future (as compared to the boxes with windows). In line with this interpretation, one theoretical perspective suggests that young children might more readily think about future possibilities than counterfactual‐past possibilities (Gautam et al. 2019; Redshaw and Suddendorf 2020), because thinking about such counterfactual possibilities requires the hierarchical understanding that in the actual past, there were alternative possible futures available (Hoerl and McCormack 2016; McCormack and Hoerl 2017; Redshaw 2024).

By contrast, when 8‐ to 9‐year‐olds were asked to directly compare the two chosen boxes, they were over four times more likely than younger children to report feeling worse about the box with a window. This clear shift away from the initial baseline tendency to choose the box without a window suggests that the older children were approaching the question in a different manner to younger children, potentially reflecting a move from a *potential‐future* orientation toward a *counterfactual‐past* orientation. Supporting this interpretation, choosing the foreseeable box was uniquely and significantly associated with the likelihood of children referencing a counterfactual in their verbal justification. Furthermore, exploratory analyses revealed that the effect of age on choosing the foreseeable box applied only to children who actively manipulated the boxes during the 15 second exploration periods, which likely reflects that these children were “closer” to the counterfactual version of the past in which they discovered the windows (see Doan et al. 2021). Nonetheless, even older children did not quite select the box with a window significantly above the chance level, perhaps indicating that many 8‐ and 9‐year‐olds continued to focus on the future functionality of the boxes.

## 4 General Discussion

Our two studies examined whether 4‐ to 9‐year‐old children accounted for the controllability and foreseeability of counterfactuals following decisions, in line with the functional theory of counterfactual thinking (Epstude and Roese 2008; Roese and Epstude 2017). Across both studies, our preregistered analyses of children's separate emotion ratings for each condition did not reveal any significant variations based on controllability or foreseeability. Only when excluding children who failed the manipulation check in Study 2, did we find that older children were significantly more likely to experience regret toward the foreseeable selection than the unforeseeable selection. Notably, however, there is evidence to suggest that children's counterfactual emotions are better assessed by asking them direct comparison questions rather than comparing separate emotion ratings between conditions (McCormack et al. 2016). Therefore, the inconsistent findings between our rating and comparison measures may reflect that children were more likely to consider controllability or foreseeability when directly prompted to make a comparison rather than spontaneously reporting different emotions one at a time.

Indeed, for the direct comparison questions, we found consistent evidence that 8‐ to 9‐year‐olds were more likely than younger children to feel worse about a more controllable event (compared to a less controllable event) that led to a negative outcome. These older children's responses were therefore increasingly in line with the functional theory of counterfactual thinking, in that they felt stronger toward the event for which they might learn to behave more adaptively in the future. The verbal justifications from Study 2 provided further support for this interpretation, as older children were more likely than younger children to report counterfactuals that would be beneficial for future decision‐making.

Focusing on the younger children only, both studies unexpectedly found that 4‐ to 5‐year‐olds reported feeling relatively worse toward a less controllable event than a more controllable event that led to a negative outcome. This pattern may reflect broader indications that children of this age typically lack the capacity to consider past counterfactuals at all (Beck 2020; Gautam et al. 2019; McCormack et al. 2019, 2020; Rafetseder et al. 2013; Redshaw and Suddendorf 2020), such that their responses in our studies were based on other aspects of controllability unrelated to counterfactuals (e.g., self‐centeredness). In Study 2, however, even 6‐ and 7‐year‐olds typically reported feeling worse about the selected box that led to an unforeseeable (i.e., less controllable) negative outcome, such that only around 10% of children of this age selected the foreseeable box with the window for the comparison question. Considering that 6‐ and 7‐year‐olds clearly show signs of counterfactual thinking in many contexts, this finding suggests that some propensities to focus on particular counterfactuals (e.g., foreseeable ones) may emerge later in development than others, and that such propensities may also differ between children and adults (see Markman and Tetlock 2000). Nonetheless, given that no children actually discovered the window mechanisms in Study 2, it remains possible that many children simply did not think they had a reasonable opportunity to foresee the actual and counterfactual outcomes. Future studies may therefore wish to manipulate foreseeability in such a manner that some children are expected to discover how to foresee the outcomes, or perhaps introduce children to a confederate who discovers how to foresee the outcomes once children's own opportunity to foresee them has passed. Under such conditions, it is plausible that even young children may be more inclined to report stronger feelings toward foreseeable than unforeseeable counterfactuals.

More generally, although we have provided some potential explanations for the age differences across studies, future research could more directly investigate which factors drive transitions in children's thinking about counterfactuals and other forms of alternative events. For example, future work could systematically test whether younger children do in fact preferentially focus on controllable future possibilities and whether older children do in fact preferentially focus on controllable past possibilities, especially in situations where thoughts about these possibilities might prompt opposite emotions. Considering that we found no age effect in the positive condition of Study 1 (and did not include a positive condition in Study 2), future research might also examine whether there are certain circumstances where older children increasingly focus on “What I could have done wrong” following a positive outcome with both controllable and uncontrollable causes (see Jones et al. 2024).

In summary, our results provide evidence that, after experiencing negative outcomes, children may not begin to shift their focus from uncontrollable to controllable factors until quite late in development. Accordingly, the propensity to reflect on more functional types of counterfactuals might emerge well after the capacity for counterfactual thinking itself.

## Supporting information


Data S1.

## Data Availability

The data and analytic code necessary to reproduce the analyses presented here have been made publicly accessible at the Open Science Framework repository. The materials necessary to reproduce the project have been displayed in figures in the main text and Supporting Information. Confirmatory analyses were preregistered. The preregistration data and analytic code for Study 1 are available at: https://osf.io/u7rjn/?view_only. The preregistration data and analytic code for Study 2 are available at: https://osf.io/q748n/?view_only.
