# Supplementary material for: The Role of Controllability and Foreseeability in Children's Counterfactual Emotions
Source: Child Dev. 2025 Feb 14;96(3):1098–111. doi: 10.1111/cdev.14224 (PMC12023811; doi:10.1111/cdev.14224)
Supplement: Supplementary file 1 — Data S1. [file CDEV-96-1098-s001.docx]

**Study 1 Supplementary Materials**

**Figure S1**

*Images of boxes from Study 1*


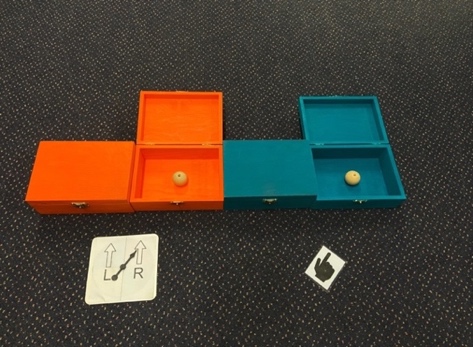

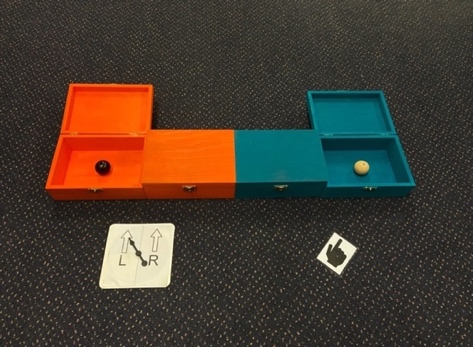


*Note:* A depiction of the task after box selection. The left panel shows an example of the winning condition, and the right panel shows an example of the losing condition.

**Figure S2**

*Post-outcome emotion ratings for each box by age group*


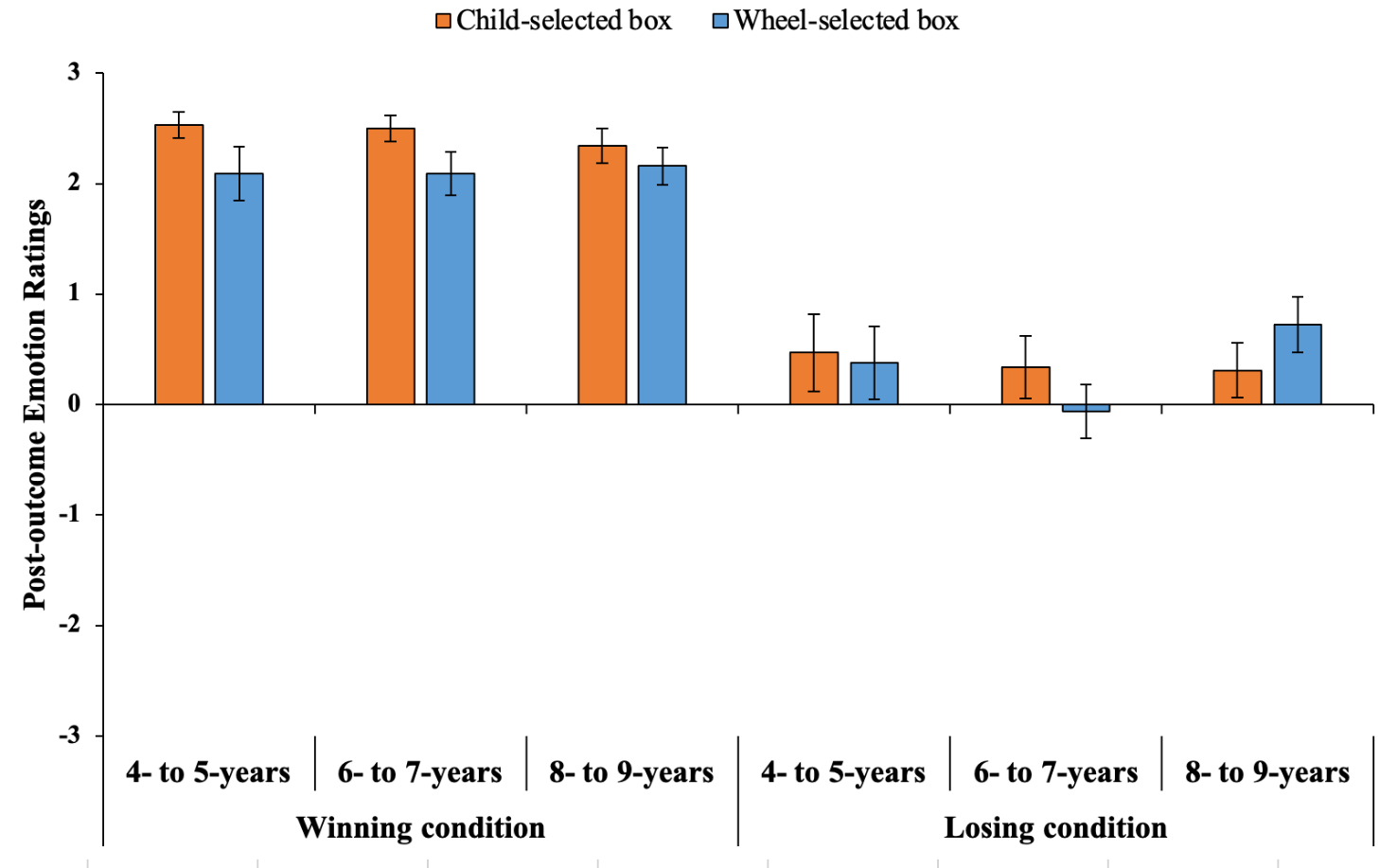


*Note:* Children’s post-outcome emotion ratings by age group, split by condition and whether the box was selected by the child (orange bars) or the wheel (blue bars). Error bars represent standard errors.

**Figure S3**

*Post-outcome emotion ratings for box selection order by age group*


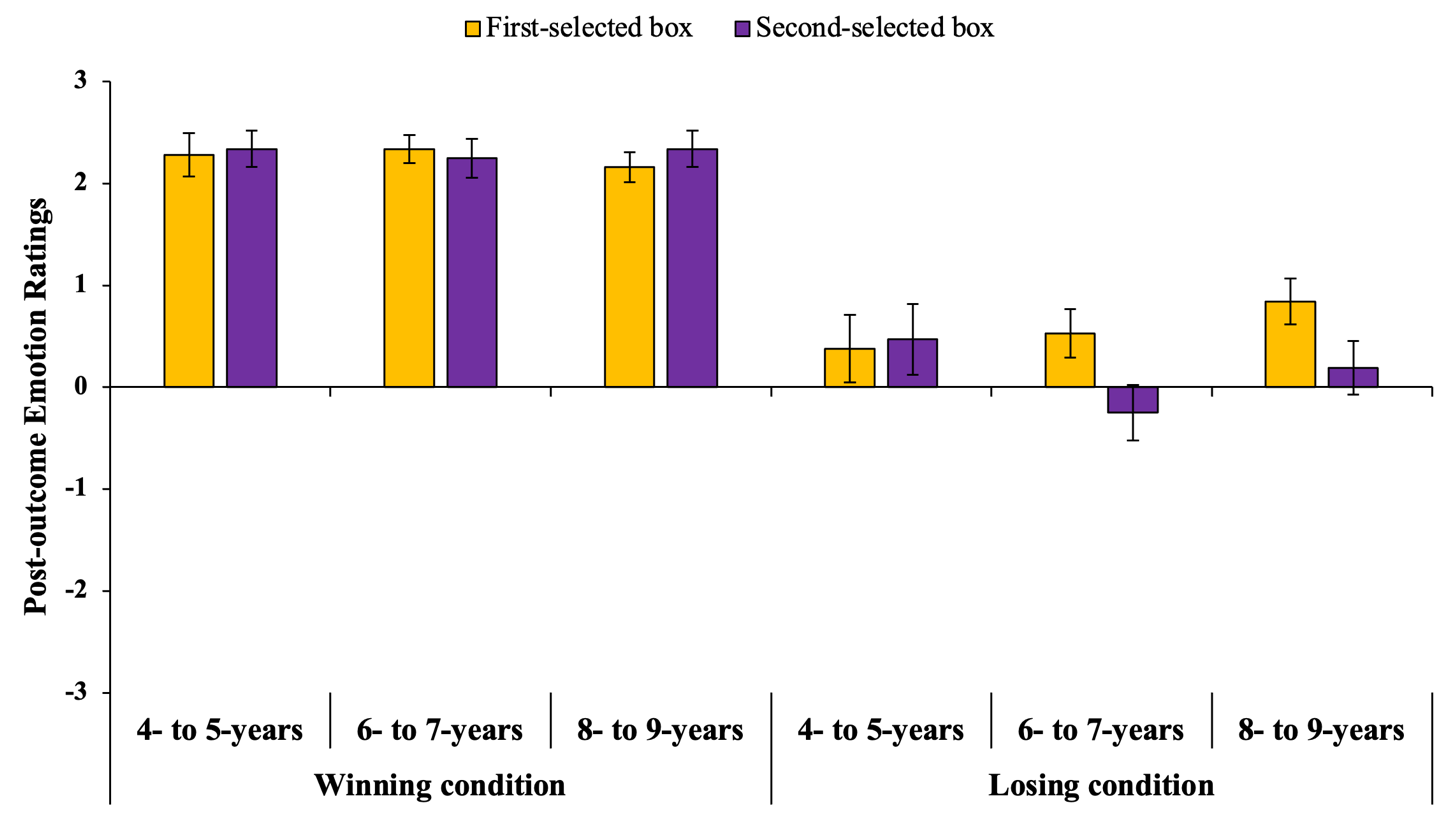


*Note:* Children’s post-outcome emotion ratings by age group, split by condition and whether the box was selected first (yellow bars) or second (purple bars), regardless of whether the box was selected by the child or wheel. Error bars represent standard errors.

**Table S1**

*Pearson’s correlations of verbal response aspects with age.*

| **Variable** | **N (out of 138)** | **Correlation with Age (*r*)** | ***p*** |
| --- | --- | --- | --- |
| Counterfactual outcome | 42 (30.4%) | .21 | .013* |
| *Winning condition (n = 71)* | 17 (23.9%) | .17 | .164 |
| *Losing condition (n = 67)* | 25 (37.3%) | .25 | .038* |
| Controllability | 41 (29.7%) | .28 | <.001** |
| *Winning condition (n = 71)* | 25 (35.2%) | .28 | **.017*** |
| *Losing condition (n = 67)* | 16 (23.9%) | .29 | **.017*** |

*Note*: *p-*values significant after Bonferroni correction (⍺ = .025) are bolded in the winning and losing conditions. ******p* <.05, ***p*<.001

**Counterfactual verbal responses analyses**

We then further coded whether the counterfactual in children’s responses (n = 42) involved reflecting on their choice or agency over the outcome. An example of a counterfactual reflecting on choice in the winning condition is “*the wheel is just chance, and I could have chosen the other box*”, and in the losing condition is “*I didn't get to choose what the wheel did, but I could have changed what I chose*”. However, an example of a response with a counterfactual not reflecting on choice in the winning condition is “*the wheel could have picked the other box*”, and in the losing condition is “*if the wheel had landed on the other box I would have won*”. Point biserial correlations revealed that counterfactuals reflecting on choice in both the winning condition (64.7%; n = 11/17) and losing condition (64%; n = 16/25) did not significantly correlate with age, *r*(15) = -.01, *p* = .958, *r*(23) = .30, *p* = .146, respectively.

An exploratory logistic regression examined whether the children who reported feeling better, in the winning condition, towards the child-selected box were more likely to refer to a counterfactual in their justification (1 = referred to a counterfactual, 0 = did not refer to a counterfactual), when controlling for age and excluding children who did not provide a verbal response. In step one, this analysis revealed children were not significantly more likely to mention a counterfactual in their response if they reported feeling better towards the child-selected box than the wheel-selected box, b = -0.47, SE = .57, Wald χ2 (1, N = 138) = 0.67, p = .413, w = 0.07. The age effect was still non-significant, b = 0.09, SE = .15, Wald χ2 (1, N = 138) = 0.30, p = .581, w = 0.04, and in step two, the age x counterfactual response interaction was not significant, b = .70, SE = .49, Wald χ2 (1, N = 138) = 1.99, p = .158, w = 0.12.

Further, we conducted the same analyses for children who reported feeling worse in the losing condition. In step one, this analysis revealed children were not significantly more likely to mention a counterfactual in their response if they reported feeling worse towards the child-selected box than the wheel-selected box, b = 0.42, SE = .57, Wald χ2 (1, N = 138) = 0.54, p = .464, w = 0.06. The age effect was weaker but still significant, b = 0.62, SE = .19, Wald χ2 (1, N = 138) = 10.65, p = .001, w = 0.28, and in step two, the age x counterfactual response interaction was not significant, b = .62, SE = .50, Wald χ2 (1, N = 138) = 1.53, p = .216, w = 0.11. Altogether, these non-significant results were likely due to the limited counterfactual responses when split into conditions (resulting in limited statistical power).

**Study 2 Supplementary Materials**

**Figure S4**

*Images of boxes from Study 2*


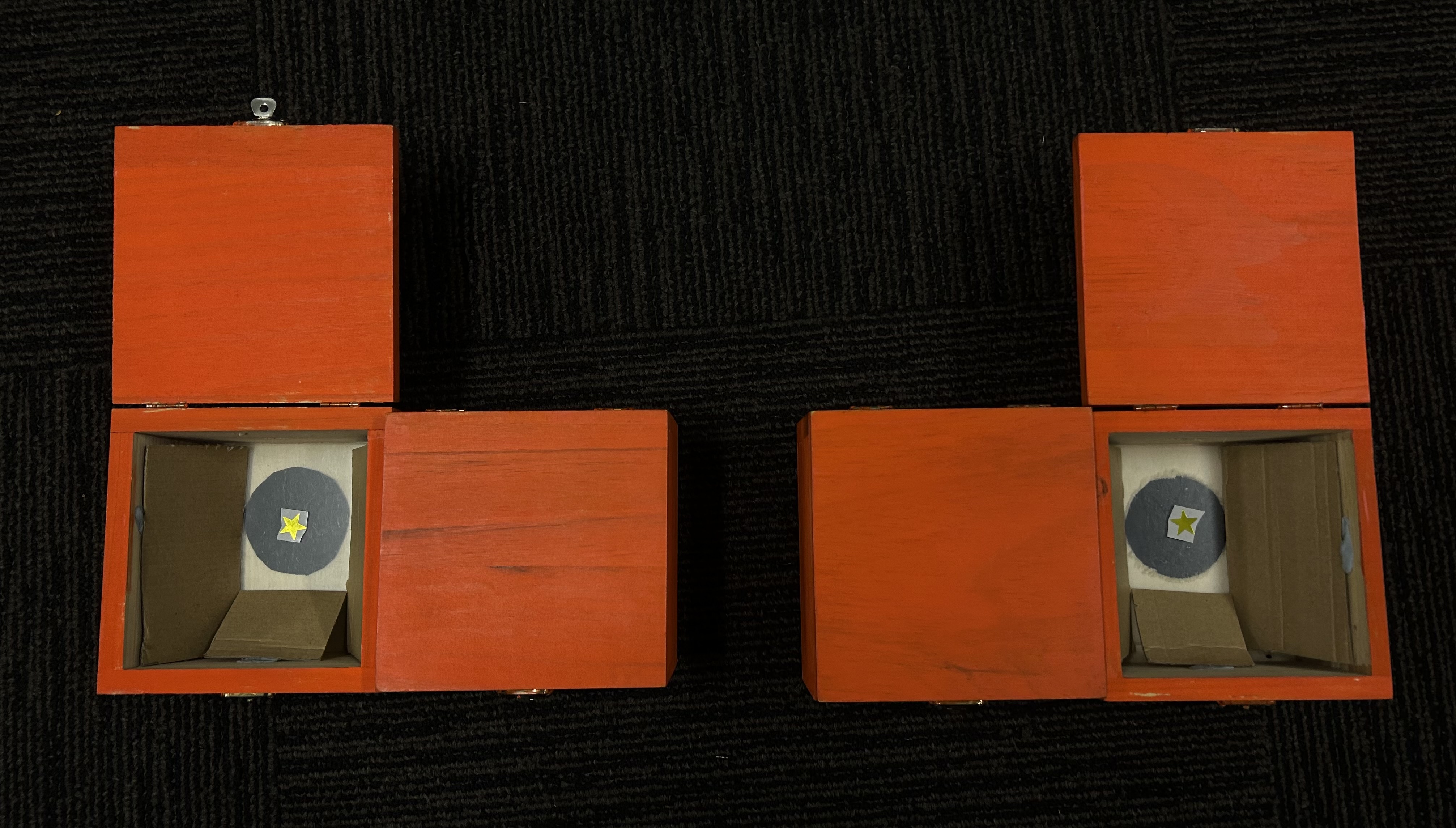

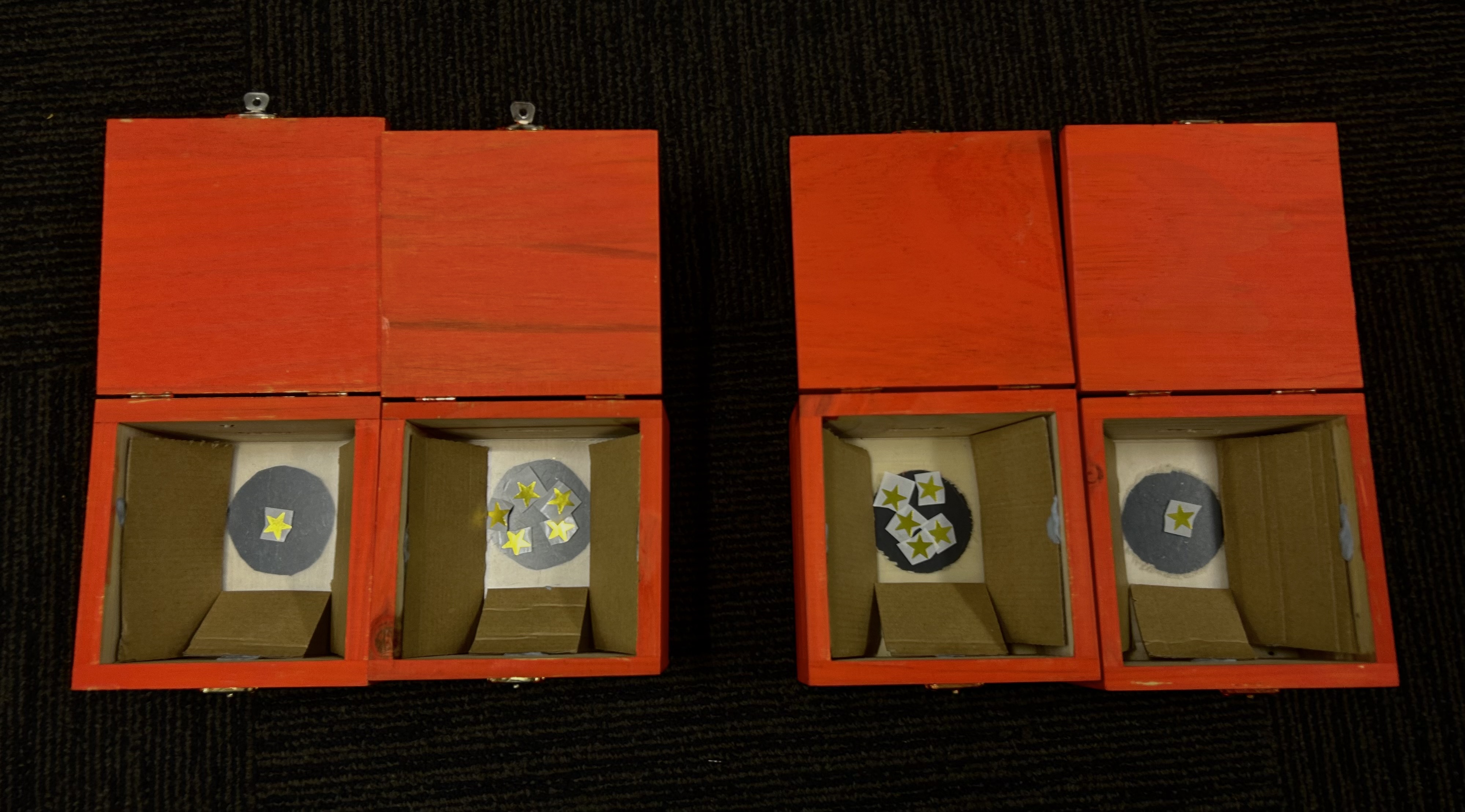

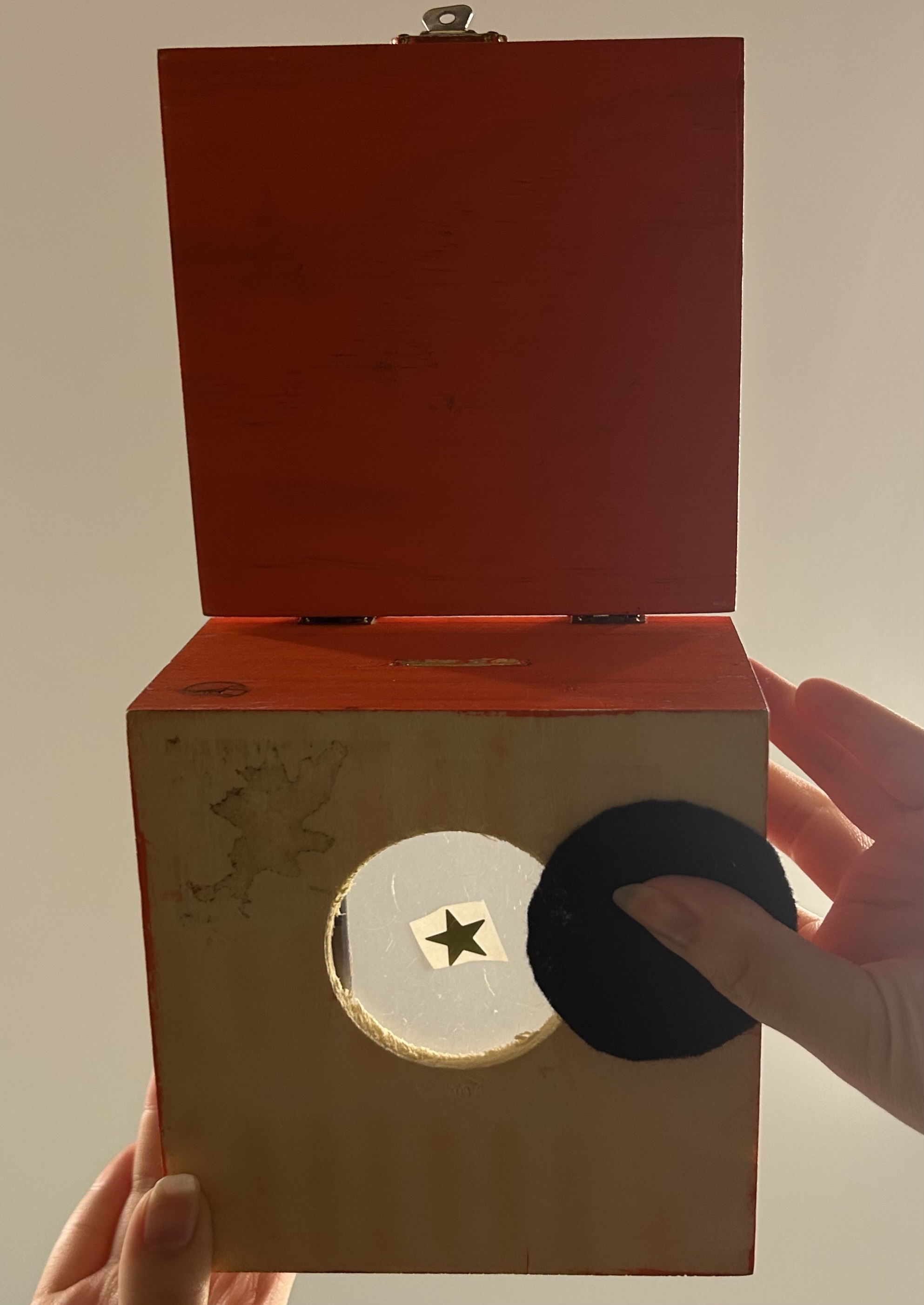

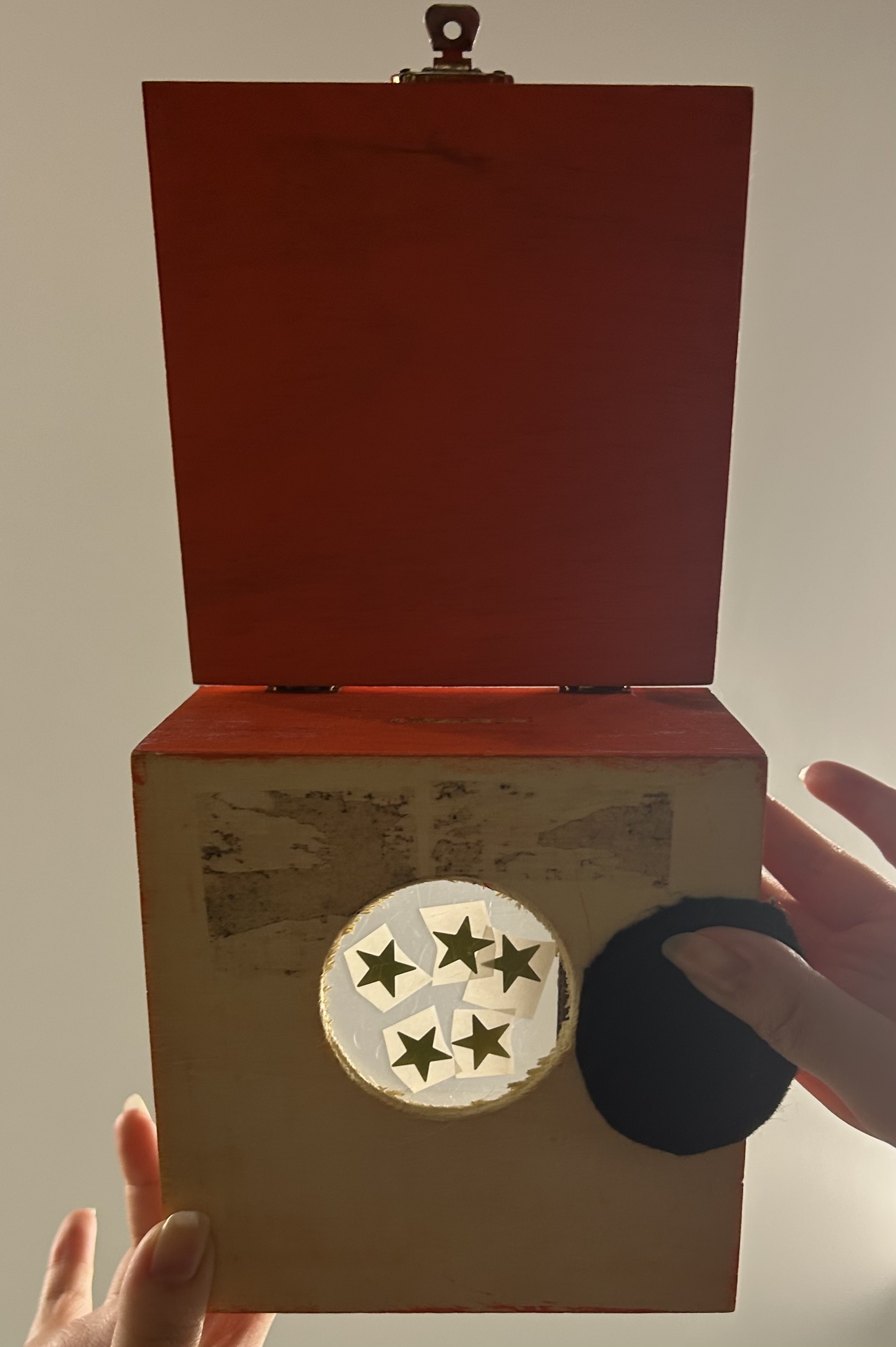


**A**

**B**

**C**

*Note:* Features of the boxes as revealed to children. (A) Both chosen boxes are first revealed to have 1 sticker each. (B) Both non-chosen boxes are then revealed to have 5 stickers each. (C) One pair of boxes is finally revealed to have windows on the bottom. The experimenter demonstrates that if you pick up these boxes and look through the bottom you can see the number of stickers inside (and also demonstrates that this is not the case for the other pair of boxes). Note that during the exploration time the lids of all boxes were closed, but the stickers were still visible through the windows of the foreseeable boxes.

**Table S2**

*Descriptives of emotion change ratings and post hoc binomial tests by age group*

| Age Group | Foreseeable box | Unforeseeable box |
| --- | --- | --- |
| 4- to 5-year-olds (*n* = 48)  *Sadder*  *The Same*  *Happier* | 10 (*p* = .042)  25 (*p* = .005*)  13 (*p* = .859) | 15 (*p* = .672)  19 (*p* = .220)  14 (*p* = .776) |
| 6- to 7-year-olds (*n* = 48)  *Sadder*  *The Same*  *Happier* | 20 (*p* = .142)  11 (*p* = .081)  17 (*p* = .432) | 19 (*p* = .220)  16 (*p* = .554)  13 (*p* = .859) |
| 8- to 9-year-olds (*n* = 48)  *Sadder*  *The Same*  *Happier* | 35 (*p* < .001*)  13 (*p* = .859)  - | 25 (*p* = .005*)  21 (*p* = .086)  2 (*p* <.001^+^) |

Note: * = significantly above chance and + = significantly below chance (33.33%, alpha = .017)

**Figure S5**

*Emotion change ratings excluding children who failed the manipulation check*

*

*Note:* Percentage of children reporting feeling sadder about the foreseeable and unforeseeable boxes after the alternative outcomes were revealed by age groups, excluding children who did not pass the manipulation check (chance level = 33.3%). **p* < .017.

**Box interaction and emotion change analyses**

An exploratory generalised linear mixed model examined whether children’s changes in emotion varied with whether children interacted with the boxes. This revealed no significant main effect of box interaction, χ2 (1, N = 123) = 0.60, *p* = .437, *w* = 0.07. There was also no significant interaction of age × box interaction, χ2 (1, N = 123) = 0.17, *p* = .683, *w* = 0.04, or foreseeability box type × box interaction, χ2 (1, N = 123) = 0.94, *p* = .333, *w* = 0.09.
